# Knowledge, attitudes and practices regarding antimicrobial use and resistance among communities of Ilala, Kilosa and Kibaha districts of Tanzania

**DOI:** 10.1186/s13756-020-00862-y

**Published:** 2020-12-07

**Authors:** Calvin Sindato, Leonard E. G. Mboera, Bugwesa Z. Katale, Gasto Frumence, Sharadhuli Kimera, Taane G. Clark, Helena Legido-Quigley, Stephen E. Mshana, Mark M. Rweyemamu, Mecky Matee

**Affiliations:** 1 National Institute for Medical Research, Tabora Research Centre, Tabora, Tanzania; 2 SACIDS Foundation for One Health, Sokoine University of Agriculture, Morogoro, Tanzania; 3 Tanzania Commission for Science and Technology, Dar es Salaam, Tanzania; 4 Muhimbili University of Health and Allied Sciences, Dar es Salaam, Tanzania; 5 College of Veterinary Medicine and Biomedical Sciences, Sokoine University of Agriculture, Morogoro, Tanzania; 6 London School of Hygiene and Tropical Medicine, London, United Kingdom; 7 Catholic University of Health and Allied Sciences, Mwanza, Tanzania

**Keywords:** Antimicrobial, Use, Resistance, Community, Knowledge, Attitude, Practices, Tanzania

## Abstract

**Background:**

Antimicrobial resistance (AMR) represents one of the biggest threats to health globally. This cross-sectional study determined knowledge, attitudes and practices (KAP) regarding antimicrobial use (AMU) and AMR among communities of Ilala, Kilosa and Kibaha in Tanzania.

**Method:**

A semi-structured questionnaire was used to collect socio-demographic and KAP data through face-to-face interviews. Responses related to the triad of KAP were assigned scores that were aggregated for each participant. Linear regression analysis was conducted to determine predictors of KAP scores.

**Results:**

The study enrolled 828 participants from the three districts. A total of 816 (98.6%) were aware of antimicrobials, and 808 (99%, *n* = 816) reported to have used them. Antimicrobials were mainly used to treat cough (68.0%), urinary tract infections (53.4%), diarrhoea (48.5%) and wounds (45.2%). The most frequent sources of antimicrobials were health facility (65.0%, *n* = 820) and pharmacies/basic drug shops (53.7%). The median AMU knowledge score was 5 (IQR = 4, 7) and that of AMR was 26 (IQR=23, 29). The median AMU attitudes score was 32 (IQR: 29, 35) and that of AMR was 19 (IQR=17, 22). The median AMU practice score was 3 (IQR: 3, 3). The KAP scores were significantly influenced by increased participant’s age (β_adj_=0.10; 95% CI: 0.05, 0.15) and level of education, being lower among those with primary education (β_adj_=5.32; 95% CI: 3.27, 7.37) and highest among those with college/university education (β_adj_=9.85; 95% CI: 6.04, 13.67).

**Conclusion:**

The study documented a moderate level of KAP regarding AMU and AMR in the study districts. The participant's age and level of education were significantly associated with participant's KAP scores. The observed inadequate knowledge, inappropriate attitude, and practices of AMU and AMR should be considered as alarming problems that require immediate actions including policy formulation and planning of community-based mitigation measures.

## Introduction

Antimicrobial resistance (AMR) has remained as one of the growing public health problem globally [1]. The misuse and abuse of antimicrobials in agriculture, veterinary, and human medicine have been described as major contributing factors for the emergence of AMR globally [2–4]. In clinical settings in Tanzania, the prevalence of multidrug-resistant bacteria ranges from 25% to 50% [5–7]. About two-thirds of isolates from wounds infections at Muhimbili National Referral Hospital were found to be resistant to at least three classes of antimicrobials [8]. Unchecked and uninformed antimicrobial use, including lack of knowledge of the course of antimicrobials, their side effects, standard acceptable dosage limits, and consequences of overdose, are the potential reasons for inappropriate or incorrect treatment or even mis-diagnosis, potentially leading to AMR [9]. Limited community knowledge, poverty, unavailability of healthcare services have also been associated with inappropriate antimicrobial use (AMU) [10–12]. In low and middle-income countries (LMIC_S_), misuse of antimicrobials has been associated with their availability over the counter, without prescription and weak regulatory frameworks [1, 13–15]. Retail pharmacies in LMIC_S_ are preferred as the primary level of patient care intensifying self-medication practices [16, 17]. Yet, the pharmacological quality of antimicrobials dispensed in these settings remains questionable [18–20]. It has been reported that one-third of the population from LMIC_S_ has inadequate knowledge about antimicrobials and their roles [21].

In LMIC_S_, the AMR is compounded by: (i) lack of access to appropriate antimicrobial therapy [16, 22]; (ii) weak regulations in the dispensing and use of antimicrobials for humans [23]; (iii) weak surveillance of AMU and AMR levels [24]; (iv) lack of updated AMU and treatment guidelines [25]; (v) lack of continuing medical/veterinary education on AMU for prescribers [26]); (vi) a weak regulatory framework for AMU in animal production and aquaculture combined with a tendency for animal owners to stock antimicrobials in their houses and engaging unskilled people such as farmers/peasants in treating animals [27]; and (vii) high degree of antimicrobial abuse by livestock keepers through failure in observing the recommended therapeutic doses, use of wrong routes of administration, arbitrary drug combinations and non-observance of withdrawing periods [28]. Other reasons include a lack of basic knowledge on the concept of antimicrobial resistance among livestock keepers [21] and unregulated disposal of waste as well as self-medication using antimicrobials [2]. AMR is being observed at the time when there has been a diminishing number of novel antimicrobials, risking the rise of untreatable infections and the inevitable loss of life [29] especially in resource-limited countries with limited treatment options.

The magnitude and consequence of AMU and misuse are unknown in many parts of Tanzania. There is a need for evidence from well-designed studies on community AMU practices to facilitate the planning and implementation of specific strategies and interventions to prevent their irrational use and hence to reduce the spread of AMR. This study was therefore carried out to determine the knowledge, attitudes, and practices regarding AMU and AMR among communities with different livelihoods in three districts of Tanzania.

## Materials and methods

### Study areas

This study was carried out in the Ilala, Kilosa, and Kibaha districts of Tanzania from January to February 2020. The districts were selected based on their demographic and ecological characteristics. Ilala district in the city of Dar es Salaam (with a highest human population density of 3,133 people per square kilometre), is characterised by urban ecosystems, occupied by multiple activities and diverse socio-ecological systems ranging from informal housing, transport infrastructure, large waste dumping sites, agriculture, industrial commercial activities, fishing and sand mining. The district is highly polluted by effluents from Msimbazi river tributaries originating from different sources, leakage of effluent from waste dumps, abattoirs and domestic wastewater from septic tanks and pit latrines that are used by about 85% of the Dar es Salaam city population. Kibaha is located in Pwani Region in eastern Tanzania (with a population density of 34 people per square kilometre) and is characterised by small-scale crop production, fish farming and large and small-scale poultry farming with frequent use of antimicrobials. Kilosa district is located in Morogoro region (with a population density of 31 people per square kilometres) in central Tanzania and is characterized by a large population of pastoralists keeping cattle, sheep and goats and known to practice self-treatment, with frequent AMU.

### Study design

This cross-sectional community-based study utilized a quantitative data collection method that was applied through a purposive and random selection process of study areas and participants. Wards were treated as the smallest spatial entities to which the targeted sample was drawn. Six wards were selected from each of the study districts using a probability proportional to human population size. Based on socio-cultural and economical values, homogeneity regarding AMU practices was assumed to exist within the members of the same household and heterogeneity was assumed between the members from different households within a ward. In this case, the number of households proportional to the targeted sample size for each ward was randomly selected from the sampling frame that was obtained from the ward office. One adult member from each of the selected household completed the face-to-face interview administered using semi-structured questionnaire installed in *AfyaData*, a digital disease surveillance app [30].

### Sample size

The pattern of AMU, and hence the risk of the onset of AMR, was assumed to vary between the three study districts and the sample size for community members to be recruited into the study was computed independently for each district. The prevalence of inappropriate AMU was estimated from studies carried out in the settings similar to each of the study districts. The prevalence of inappropriate AMU was estimated at 71.5% [31], 90% [32], and *7*4% [33], for Ilala, Kibaha and Kilosa, respectively. Assuming the finite population and relative precision of 5% at a 95% confidence interval, the minimum sample size was estimated to be 314, 139 and 296 for Ilala, Kibaha and Kilosa, respectively. Assuming a 10% dropout or refusal rate, the resulting minimum sample size for Ilala, Kibaha and Kilosa was 345, 153 and 326, respectively. The targeted sample size for each district was divided equally into six wards.

### Data collection

Before the commencement of data collection exercise, twelve research assistants were trained on the study objectives, study protocol, sampling strategy, community engagement and ethical issues. They were provided with visual aids including pictures of antimicrobials to make interactions with study participants clear or easy to understand. A pre-tested questionnaire was used to collect all data through face-to-face interviews. The data collected included socio-demographic characteristics (age, sex, occupation, village, workplace, and residence), knowledge of antimicrobials (what are they and their roles, types of antimicrobials known and health conditions treatable with antimicrobials), practices of AMU (preferences, frequency of use, sources, ill-health conditions treated, adherence to the treatment regimen, self-medication practices). We also assessed awareness and knowledge of AMR. In addition to dichotomous responses to some questions, the five-point Likert scales (‘strongly agree’, ‘agree’, ‘uncertain’, ‘disagree’ and ‘strongly disagree’) were used to determine the participants’ knowledge and attitudes regarding antimicrobials, their uses as well as the burden, actions and roles to address AMR.

### Data management and analysis

The data collected from households were submitted on daily basis to a server located at Sokoine University of Agriculture, Morogoro Tanzania. After data collection, it was exported from the server to Excel spreadsheet for coding before been imported to Stata version 13 (Stata Corp., College Station, TX, USA) for cleaning, descriptive and statistical analysis. Responses to the triad of knowledge, attitudes and practices (KAP domains) were assessed using a scoring scheme. For dichotomous responses, a right response was given one score, and zero to the wrong/unanswered option. Multiple responses to each of the correct options were given one score; otherwise, no score was given [34]. Responses to the five-point Likert scale were given scores that ranged from “5” for the most appropriate answer to “1” for the least appropriate answer and summed to form a discrete variable [35]. The AMU knowledge scores ranged from 0 to 13, the AMR knowledge scores from 0 to 40, the AMU attitude scores ranged from 0 to 40, the AMR attitude scores from 0 to 25 and that of AMU practice from 0 to 7 points.

The median total score of responses to questions related to each of the three KAP domains was calculated together with its interquartile range (IQR) presented as the first and third quartiles. The strength of associations between socio-demographic variables and knowledge, attitudes and practices regarding AMU and AMR was measured using Chi-square tests. The Levene's test for variance indicated a statistically significant difference in the variance of some of the scores across socio-demographic variables. For this reason, a pairwise test using “rank-sum” test was used with caution to test the difference of the median scores. For interpretation of the analysis outputs from the scoring scheme, a score greater than 80% of the possible maximum scores was considered good, between 60% and 80% were considered moderate and less than 60% was considered poor [35].

The influence of socio-demographic factors on participants’ knowledge, attitude and practice scores were analysed using both bivariate and multivariate linear regression models. The null hypothesis tested was that the predictor coefficients are 0, and the alternate hypothesis was that the coefficients are non-zero. First, the association between socio-demographic variables (independent variables) and each of the three KAP domains was screened for statistical significance at a p-value of ≤ 0.20 in bivariate analysis. From the bivariate analysis, the statistically significant variables were included in a multivariate linear regression analysis based on a forward variable selection approach utilising the likelihood ratio statistic and a p-value ≤ 0.05. The results of bivariate and multivariate analysis were reported as regression coefficient beta (β) and adjusted coefficient beta (β_adj_), respectively together with their corresponding confidence intervals (95% CI) at a p-value of 0.05. The variables included in the modelling process were limited to those that did not show significant collinearity using a diagnostic cut-off value for tolerance > 0.1 and variance inflation factor < 10 [36]. The Spearman rank-order correlation coefficient (rho) was run to assess the relationship and direction of the association between each pair of knowledge, attitudes and practices scores for participants.

## Results

### Socio-demographic characteristics

A total of 828 participants from Kilosa (360, 43.5%), Ilala (312, 37.7%) and Kibaha (156, 18.8%) were involved in the study. Females accounted for about three-quarter (73.1%) of participants. The overall median age of participants was 40 years (IQR: 30, 52). The median age of females and males was 38 (IQR: 29, 50) and 45 (IQR: 34, 58), respectively. In all the study districts, almost two-thirds of the participants were married. Overall, over half (59.4%) had attained primary school education; 18.4%-secondary; and 4.4%-college/university education. Agriculture (growing crops and raising livestock) and business, each accounted for over a third of the main sources of income to participants. While almost three-quarter (73.7%) of participants from Ilala reported business as the main source of income, majority of those from Kilosa (57.2%) and Kibaha (46.8) reported agriculture (Table 1).

**Table 1:** Socio-demographic characteristics of participants from Ilala, Kilosa and Kibaha

Variable	Ilala (***n*** = 312)	Kilosa (***n*** = 360)	Kibaha (***n*** = 156)
Sex (%)			
Female	229 (73.4)	252 (70.0)	124 (79.5)
Male	83 (26.6)	108 (30.0)	32 (20.5)
Age in years			
Median (IQR)	38 (29, 50)	40 (30, 54)	40 (29, 53.5)
Marital status (%)			
Single	58 (18.6)	43 (11.9)	16 (10.3)
Married/cohabiting	222 (71.2)	256 (71.1)	109 (69.9)
Widower/widow	18 (5.8)	40 (11.1)	21 (13.5)
Divorced/separated	14 (4.5)	21 (5.8)	10 (6.4)
Formal educational level attained (%)			
No formal education	13 (4.2)	107 (29.7)	28 (18.0)
Primary	173 (55.5)	184 (51.1)	92 (59.0)
Secondary	91 (29.2)	37 (10.3)	24 (15.4)
College	27 (8.7)	3 (0.8)	1 (0.6)
University	4 (1.3)	1 (0.3)	0 (0.0)
Main source of income			
Agriculture	4 (1.3)	206 (57.2)	73 (46.8)
Employment	75 (24.0)	25 (6.9)	19 (12.2)
Petty trading	233 (74.7)	129 (35.8)	64 (41.0)

### Awareness and knowledge of antimicrobial use and resistance

Of the 828 participants, 816 (98.6%) were aware of antimicrobials. The level of awareness was significantly higher for participants from Kibaha (100%, *n* = 156) and Ilala (99.7%, *n* = 312) than those from Kilosa (96.9%, *n* = 360) (*p* = 0.003). The level of awareness tended to increase slightly with an increased level of education. The majority (95.3%) of those with no formal education (*n* = 148) reported lower level of awareness than those with primary (99.0%, *n* = 492), secondary (100%, *n* = 152) and college/university (100%, *n* = 36) level of education (*p* = 0.002). The higher level of awareness was recorded among participants whose main source of income was petty trading (99.8%, *n* = 449) and agriculture (99.8%, *n* = 283) than those who were employed (92.7%, *n* = 96) (*p* < 0.001). The level of awareness was similar between males and females and did not vary with age (*p* > 0.05). Out of 816 participants who reported on awareness of antimicrobials, the majority (97.6%) associated AMU with the treatment of diseases. Other reported uses of antimicrobials included prevention of diseases (13.3%), growth promotion (0.9%) and feed additives for animal production (0.6%). The types of antimicrobials that were known to participants included amoxicillin (66.0%), tetracycline (23.8%), metronidazole (20.7%), ampicillin (14.9%), ampiclox (7.5%), ciprofloxacin (5.2%), penicillin (4.2%), erythromycin (3.6%) and doxycycline (2.3%).

**Fig. 1:**
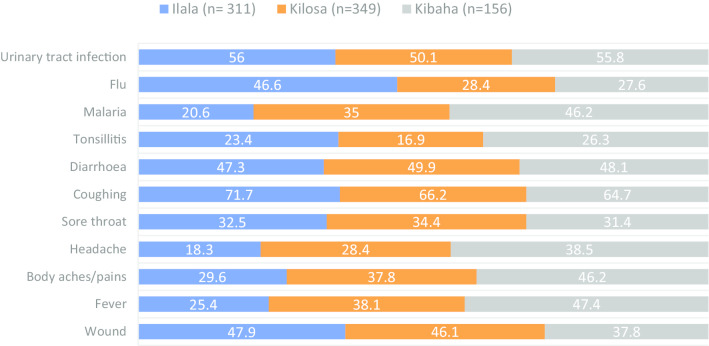
Distribution of the proportion of participants on their awareness of disease conditions treatable with antimicrobials by district

Participants frequently mentioned coughing (68.0%), urinary tract infections (53.4%), diarrhoea (48.5%) and wounds (45.2%) as the health conditions frequently treated by antimicrobials. A significantly higher proportion of participants from Kibaha than those from other study districts reported fever (47.4%, *p* < 0.001), body aches/pains (46.2%, *p* = 0.002), malaria (46.2%, *p* < 0.001) and tonsillitis (26.3%) as the conditions treatable with antimicrobials. On the other hand, a significantly larger proportion of participants from Ilala (46.6%, *p* < 0.001) than those from other districts reported cold, flu and runny noses as the conditions treatable with antimicrobials (Fig. 1).

The overall median score of AMU knowledge in the three districts was 5 (IQR=4, 7). A relatively higher value of median score of 6 (IQR: 4, 7) was observed for participants from Kibaha than from Kilosa (median=5, IQR: 4, 7) and Ilala (median=5; IQR: 4, 7) (*p* = 0.0065). The median score of knowledge tended to increase with an increased level of education as demonstrated by higher value recoded in participants with college/university level of education (median=7; IQR: 5, 8) than those with secondary (median=6; IQR: 4, 7), primary (median=5; IQR: 4, 7) and those with no formal education (median=4; IQR: 3, 6) (*p* = 0.0001). Overall, 84.9% and 15.1% of participants (*n* = 816) had a poor and moderate levels of antimicrobial knowledge, respectively.

### Knowledge of antimicrobial resistance

Over half of participants from Ilala (56.3%) and less than half from Kilosa (40.4%) and Kibaha (35.9%) strongly agreed that AMR occurs when antimicrobials no longer able to treat infections (*p* < 0.001). This level of agreement was more evident among participants with secondary and college/university than those with a lower level of education (*p* = 0.006). About half (52.0%) of participants whose main source of income was from petty trading strongly agreed with the statement compared with those whose main source of income was agriculture (38.0%) or employment (37.1%) (*p* < 0.001). Similarly, about half (51.8%) of participants from Ilala and about one-third from Kilosa (35.2%) and Kibaha (32.1%) strongly agreed that many infections are becoming increasingly resistant to treatment by antimicrobials (*p* < 0.001). This level of agreement was observed more frequently among participants with secondary and college/university than those with lower levels of education (*p* < 0.001). Likewise, a relatively larger proportion (46.7%) of participants whose main source of income was from petty trading strongly agreed with the statement than those who were employed (34.8%) (*p* < 0.001) and those whose main source of income was from agriculture (33.7%) (*p* < 0.001).

In Ilala, over half (54.7%) of participants strongly agreed that if the disease-causing germs are resistant to antimicrobials, it can be very difficult or impossible to treat the infection they cause. About one-third of participants from Kilosa (36.1%) and Kibaha (34.0%) expressed a similar level of agreement (*p* < 0.001). Males were more likely to strongly agree with the statement than females (*p* = 0.001). A similar pattern of the agreement was observed among participants aged >33 years old than among the younger ones (*p* = 0.026). Over two-thirds (69.4%) of participants with college/university level of education compared with those with secondary (45.4%), primary (43.5%) levels and those with no formal education (30.5%) (*p* < 0.001) strongly agreed that it is difficult to treat diseases caused by resistant pathogens. There was some statistical evidence to suggest that being from a petty trading category of occupation (47.8%) was more likely associated with a tendency to strongly agree with the statement compared with being from employment (38.2%) or agriculture (36.2%) (*p* = 0.013) category.

Over half (55.6%) of participants from Ilala and less than half from Kibaha (46.2%) and Kilosa (34.1%) strongly agreed that AMR is mainly a problem for people who take antimicrobials frequently (*p* < 0.001). Over half (58.3%) of participants with college/university level of education and less than half of those with lower levels of education expressed the same agreement (*p* = 0.001). About half (50.9%) of participants from petty trading category and just over one-third of those who were employed (38.2%) and those working in agriculture (36.6%) expressed the same agreement. Only about one-third of participants from Kilosa (36.1%), Ilala (34.7%) and Kibaha (34.0%) strongly agreed that disease-causing germs that are resistant to antimicrobials can be spread from person to person (*p* = 0.002).

The overall median score of AMR knowledge in the three districts was 26 (IQR=23-29) but differed significantly between the sociodemographic variables. Participants from Ilala demonstrated a higher value (median=27; IQR: 23, 21) than those from Kibaha (median=26; IQR: 23, 29) and Kilosa (median=25; IQR: 22, 29) (*p* = 0.0003). The median score of knowledge on AMR increased with an increase in the level of education. A higher value was recorded in participants with college/university level of education (median=28.5; IQR: 26, 31) than those with secondary (median=27; IQR: 23, 30), primary (median=26; IQR: 23, 29) or those without formal education (median=25; IQR: 22, 28) (*p* = 0.0001). Participants whose major source of income was from petty trading had a higher median score of 27 (IQR: 23-30) than those who were employed (median=25, IQR: 22, 29) and agriculture keeping (median=25, IQR: 22, 29) (*p* = 0.0008). Males had significantly higher scores (median=27; IQR: 24, 31) than females (median=26, IQR: 23, 29) (*p* = 0.0022). About two-thirds (62.5%; *n* = 816) of the participants had moderate knowledge of AMR, 29.5% had poor knowledge and 8.0% had good knowledge.

### Attitudes on the appropriate use of antimicrobials

Participants were presented with different statements related to the use of antimicrobials and asked on how much they would agree or disagree with them. More positive views largely accorded with the strongly agree category, as did the less positive with the disagree category. A significant difference was observed regarding attitudes. A larger proportion of participants from Kilosa (84.5%), Ilala (83.6%) and Kibaha (73.7%) strongly agreed that people should use antimicrobials only when they are prescribed by the health care professionals (*p* = 0.002). Washing hands was considered one of the measures to prevent the spread of disease-causing germs and thus reduce AMU. When asked about this relationship, the largest proportion of participants who strongly agreed that people should wash their hands regularly were from Ilala (93.9%) followed by Kibaha (85.1%) and Kilosa (85.1%) (*p* < 0.001). On the other hand, more positive views that largely accorded with strongly agree category concerning health care professionals only prescribing antimicrobials when they are needed, were recorded in Ilala (87.5%) followed by Kilosa (73.1%) and Kibaha (62.2%) (*p* < 0.001). On the contrary, rather few views that accorded with the strongly agree category regarding people not keeping antimicrobials and use them for other illnesses, was recorded in Ilala (58.5%), followed by Kibaha (44.2%) and Kilosa (43.0%) (*p* < 0.001).

The overall median score of attitudes regarding appropriate AMU in the study districts was 32 (IQR: 29, 35). The attitude varied significantly between the districts, with higher values demonstrated by participants from Ilala (median=33; IQR: 30, 35) followed by Kilosa (median=32; IQR: 29, 35) and Kibaha (median=30; IQR: 27, 33) (*p* = 0.0001). Participants with college/university (median=33; IQR: 30, 35) and primary (median=33; IQR: 29, 35) levels of education expressed better attitude than those with secondary (median=32; IQR: 29, 35) and those without formal education (median=31; IQR: 28, 34) (*p* = 0.0007). Over two-third (69.9%, *n* = 816) of participants demonstrated a moderate attitude on the appropriate use of antimicrobials, 22.4% good attitude and 7.7% poor attitude.

### Attitudes on the burden of AMR

About half of participants from Ilala (54.0%), Kibaha (51.3%) and less than half from Kilosa (41.2%) strongly agreed that AMR is one of the biggest problems the world is facing currently (*p* < 0.001). Furthermore, almost two-thirds (64.1%) of participants from Kibaha strongly agreed that Tanzania is among the countries facing the challenges related to AMR. On the contrary, less than half of the participants from Ilala (43.7%) and Kilosa (38.4%) had expressed a similar level of agreement (*p* < 0.001). The pattern of agreement with the statement tended to vary with the level of education as it was observed in over three-quarter (86.1%) of participants with college/university level of education compared with those with secondary (59.9%), primary (55.7%) or with no formal education (44.0%) (*p* = 0.004). A relatively larger proportion (60.0%) of participants from the petty trading category of occupation expressed the same agreement compared with those from agriculture (50.9%) or employment (49.4%) categories (*p* = 0.030).

About three-quarters of participants from Ilala (70.1%) and a half from Kilosa (55.6%) and Kibaha (53.9%) strongly agreed that medical experts could resolve the problem of AMR before it becomes too serious (*p* = 0.001). While a greater proportion of participants from Ilala (71.4%) and Kilosa (61.3%) strongly agreed that everyone needs to use antimicrobials responsibly, those from Kibaha (44.2%) were less likely to have a similar opinion (*p* < 0.001). Approximately, one third of participants from Kibaha (30.7%), Kilosa (33.9%) and Ilala (37.6%) were of the opinion that they could at least do something to stop AMR (*p* < 0.001). While over half (56.0%) of participants from Ilala were strongly worried about the impact that AMR will have on their health and that of their families, those from Kibaha (41.7%) and Kilosa (39.5%) were less likely to have same opinion (*p* < 0.001). Similarly, while over half (57.6%) of participants from Ilala strongly agreed that they were not at risk of getting AMR infections, as long as they take antimicrobials correctly, fewer participants from Kilosa (45.0%) and Kibaha (36.5%) were likely to express a similar opinion (*p* < 0.001). Majority of participants from Ilala (81.7%), and about two-third from Kilosa (67.6%) and Kibaha (65.4%) strongly disagreed with the statement that it is appropriate to use antimicrobials that were given to a friend or family member, as long as they were used to treat the same illness (*p* < 0.001). About half (52.1%) of participants from Ilala, strongly disagreed with the statement that it is appropriate to buy the same antimicrobials, or request these from a doctor, if you are sick and they have helped you get better when you had the same symptoms before. However, relatively low proportions of participants from Kilosa (44.1%) and Kibaha (43.0%) were likely to express the same opinion (*p* < 0.001).

The overall median score of attitude regarding the burden of AMR and individual roles to address the problem was 19 (IQR=17, 22). The distribution of the median scores differed significantly between the sociodemographic variables. Participants from Ilala demonstrated higher values (median=20, IQR: 17, 23) than those from Kilosa (median=19 (IQR: 16, 21) and Kibaha (median=18.5 (IQR: 16, 21) (*p* = 0.0018). The median score tended to increase with an increased level of education. Higher values were recorded in participants with college/university level of education (median=20.5; IQR: 19, 23) than those with secondary (median=20; IQR: 17, 22), primary (median=19; IQR: 17, 22) and no formal education (median=18; IQR: 16-20) (*p* = 0.0001). Participants whose major income was from agriculture (median=19; IQR: 16, 21) and petty trading (median=19; IQR: 17, 22) had higher median scores than those whose major source of income was from employment (median=17, IQR: 15, 21) category (*p* = 0.0001). About half (52.7%, *n* = 816) of participants expressed moderate attitude regarding the burden of AMR and individual role to addressing the problem, over one-third (37.5%) and 9.8% showed good and poor attitudes, respectively.

### Practices on use of antimicrobials

**Fig. 2:**
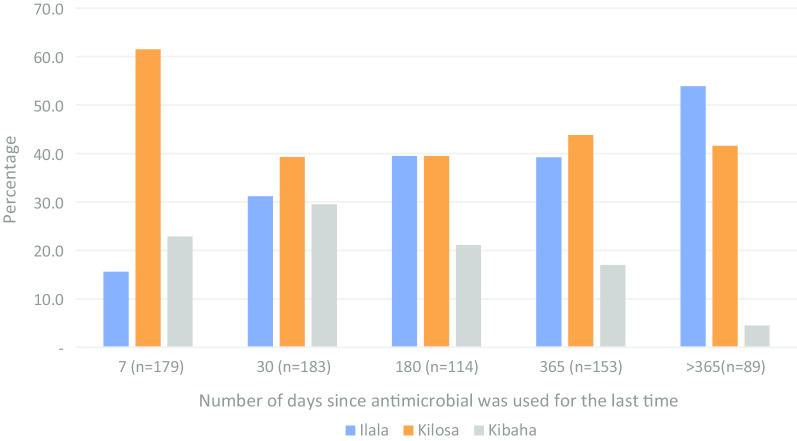
Percentage distribution of participants’ history of antimicrobials use by district

**Fig. 3:**
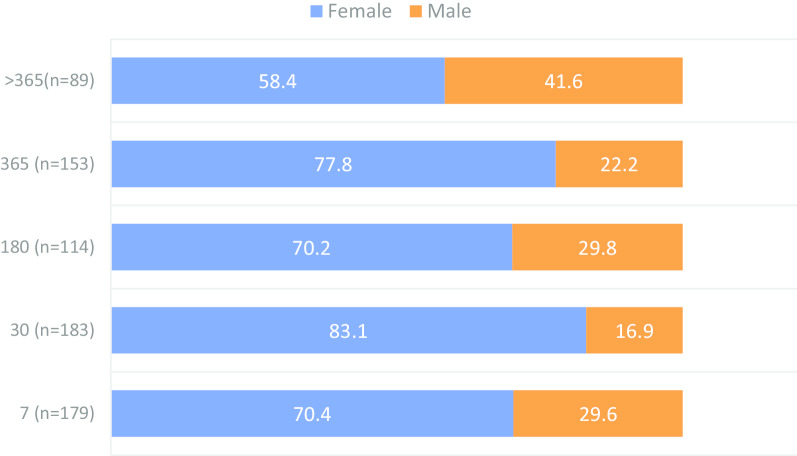
Percentage distribution of participants’ history of antimicrobial use by sex

Overall, almost all (99.0%, *n* = 816) participants who reported awareness on antimicrobials, affirmed to have ever used antimicrobials in their lifetime. They represented all participants from Kilosa and Kibaha, and 97.4% of participants from Ilala (*p* = 0.001). Of the 808 participants who reported to have ever used antimicrobials, 718 (88.9%) could recall the last time they had used antimicrobials. Specific periods of the history of AMU was organized into five categories, which were the past 7, 30, 180, 365 and >365 days. When participants were asked to recall the last time they took antimicrobials, a significant difference was observed between the districts and sex (*p* < 0.05). Overall, the recent AMU was frequently reported among participants in Kilosa and Kibaha than Ilala. Almost two-thirds (61.5%) of participants who reported to have ever used antimicrobials during the past 7 days (*n* = 179), were from Kilosa (Fig. 2). Throughout the recall periods, females were consistently on the higher frequency of AMU than males (*p* = 0.001) (Fig. 3). Besides the observed association between age and preference to antimicrobials, the actual AMU tended to be homogenously distributed across age groups (*p* > 0.05). A similar pattern was observed for the association between the AMU and level of education, marital status or occupation (*p* > 0.05).

The overall median score of appropriate AMU was 3 (IQR: 3, 3). All three districts had the same median value. Participants from Kibaha and Kilosa had the same IQR (3, 3) while those from Ilala had IQR of 3, 4 (*p* = 0.0086). Similarly, the same median of 3 was observed across different levels of education and type of occupation, with slight differences in the IQRs. Participants with college/university and secondary level of education had the same IQR (3, 4) while those with primary (3, 3) and with no formal education (2, 3) had slightly less (*p* = 0.0086). Those whose main source of income was agriculture and petty trading had relatively higher IQR (3, 3) than those from employment category (2, 3) (*p* = 0.0253). The results showed that almost all (97.3%, *n* = 816) had a poor practice of AMU, 2.8% had moderate practice and 0.1% had a good practice.

### Preference for antimicrobials

**Fig. 4:**
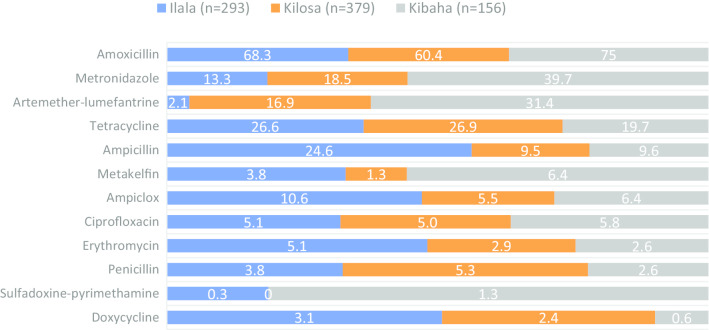
Distribution of the proportion of participants on their antimicrobial preferences by district

Amoxicillin was the most frequently preferred antimicrobial in all the three districts (Kibaha=75.0%; Ilala=68.3%; Kilosa=60%) (*p* = 0.002). A significantly larger proportion of participants from Kibaha had more preference for metronidazole (39.7%) than those from Kilosa (19.2%) and Ilala (12.8%) (*p* < 0.001). A higher proportion of participants from Kilosa (26.9%) and Ilala (23.7%) preferred tetracycline than those from Kibaha (19.7%) (*p* = 0.004). A significantly larger proportion of participants from Ilala (24.0%) reported a preference for ampicillin than those from Kibaha (9.6%) and Kilosa (9.2%) (*p* < 0.001). Only a few (<10%) participants from Ilala, Kilosa and Kibaha had a preference to ciprofloxacin, erythromycin, penicillin or ampicillin/cloxacillin (Fig. 4).

Almost three-quarter of females (71.9%, *n* = 605) had preference to amoxicillin compared to males (49.8%, *n* = 223) (*p* = 0.001). A significant variation was observed on females’ than males’ preference to amoxicillin in Ilala and Kilosa (*p* < 0.001). Sex was not significantly associated with preference to this antimicrobial among respondents in Kibaha. Relatively larger proportion of participants aged 18-33 years old (74.4%, *n* = 282) and 34-47 years old (66.6%, *n* = 275) had preference to amoxicillin than those aged 48-95 years old (56.6%, *n* = 272) (*p* < 0.01). When the analysis was controlled by the district, a similar pattern of significant variation in the association between age categories and preference to amoxicillin was maintained among participants from Ilala (*p* = 0.005) and Kibaha (*p* = 0.001) with insufficient evidence of statistical difference among participants from Kilosa (*p* > 0.05).

### Sources and factors influencing the choice of antimicrobials

**Fig. 5:**
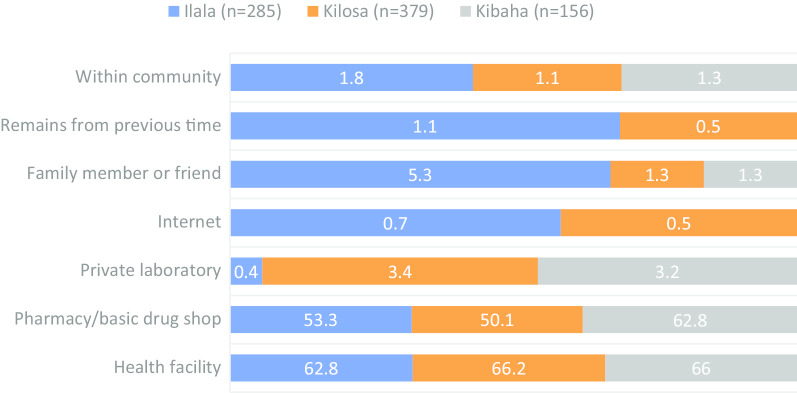
Sources of antimicrobials frequently reported by participants by district

Overall, the most frequent sources of antimicrobials were health facility (65.0%, *n* = 820) and pharmacies/basic drug shops (53.7%) (Fig. 5). A relatively higher proportion of participants from Kibaha (62.8%, *n* = 156) were more likely to obtain antimicrobials from pharmacies/basic drug shops than those from Ilala (53.3%, *n* = 285) and Kilosa (50.1%, *n* = 379) (*p* = 0.028).

Overall, two-thirds (65.5%, *n* = 542) of participants reported that their choice of antimicrobial was influenced by advice from health care providers. Nearly a quarter (23.3%) of participants had their choice influenced by advice from pharmacists, and this varied significantly between the districts. A relatively larger proportion of individuals from Kibaha (37.8%, *n* = 156) were more likely to rely on pharmacist's advice than those from Kilosa (20.1%, *n* = 379) or Ilala (19.8%, *n* = 293) (*p* < 0.001). Less than a quarter (17.4%) of all participants had their choice of antimicrobials influenced by experience from the previous episode. A relatively larger proportion of individuals from Ilala (24.2%) had their choice driven by experience from the previous episode followed by those from Kilosa (14.8%) and Kibaha (10.9%) (*p* < 0.001).

**Fig. 6:**
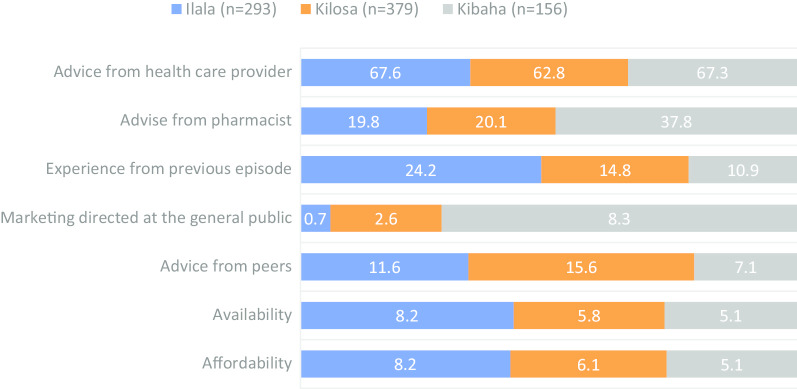
The proportion of respondents as regards to factors influencing the choice of antimicrobials in by the district

Although only 3.0% of individuals were likely to choose antimicrobials based on availability in the general public markets, there was statistical evidence to suggest that the practise was more frequent among participants from Kibaha (8.3%) than those from Kilosa (2.6%) or Ilala (0.7%) (*p* < 0.001). There was weak statistical evidence to suggest that individuals from Kilosa (15.6%) and Ilala (11.6%) were more likely to seek advice from their peers regarding the type of antimicrobial to use than those from Kibaha (7.1%) (*p* = 0.022). Availability of antimicrobials and individuals’ affordability appeared to be the weak drivers for antimicrobial choice (Fig. 6). Individuals aged 48-95 years old (72.1%, *n* = 272) were more likely to seek advice on antimicrobial to use from health care providers than their younger counterparts (*p* = 0.017). Individuals who were not married and those who had separated were less likely to seek advice from health care worker regarding the type of antimicrobials to use (*p* = 0.038).

### Adherence to the treatment regimen

Overall, more than three-quarters (80%, *n* = 808) of individuals who reported to have ever used antimicrobials, were likely to complete a full course of treatment. Less than a quarter (19.4%) reported that they would not complete the treatment regimen when they feel better after a three-day treatment. A relatively higher proportion of individuals from Ilala (19.5%, *n* = 312) were more likely to keep the leftover antimicrobials for future use compared with those from Kibaha (15.4%, *n* = 156) and Kilosa (11.1%, *n* = 360) (*p* = 0.01). Likewise, a relatively higher proportion of individuals from Ilala (18.6%) were more likely to dispose the leftover medicines compared with those from Kibaha (6.4%) and Kilosa (3.9%). Socio-demographic factors were found to have no significant influence on adherence to the treatment regimen (*p* > 0.05).

### Antimicrobial prescription and self-medication practices

**Fig. 7:**
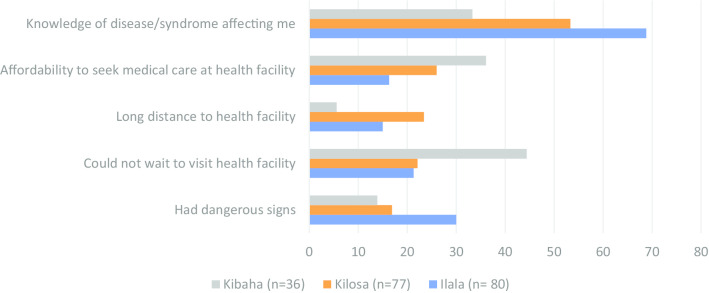
Reasons for self-medication by district

Overall, about three-quarter (76.4%, *n* = 808) of participants reported that it was a common practice to use antimicrobials with a prescription from the health care worker or pharmacist. When they were asked about the practice during the most recent time they had used antimicrobials; overall, 74.4% of reported to have used antimicrobials with a prescription. The most frequent reasons for self-medication practices were knowledge of the disease/syndrome affecting an individual (56.0%), individual not been able to wait until she/he is served at a healthcare facility (25.9%), affordability to seek medical care (23.8%), suffering from dangerous signs (21.8%) and distance to the healthcare facility (16.6%) (Fig. 7). The knowledge of the disease condition affecting an individual varied between the districts with significantly greatest proportion been recorded from Ilala (68.8%, *n* = 80) followed by Kilosa (53.3%, *n* = 77) and Kibaha (33.3%, *n* = 36) (*p* < 0.001). Likewise, individuals from Kibaha were more likely to express not been able to wait to visit a healthcare facility (44.4%) than those from Kilosa (22.1%) or Ilala (21.3%) (*p* = 0.019).

**Fig. 8:**
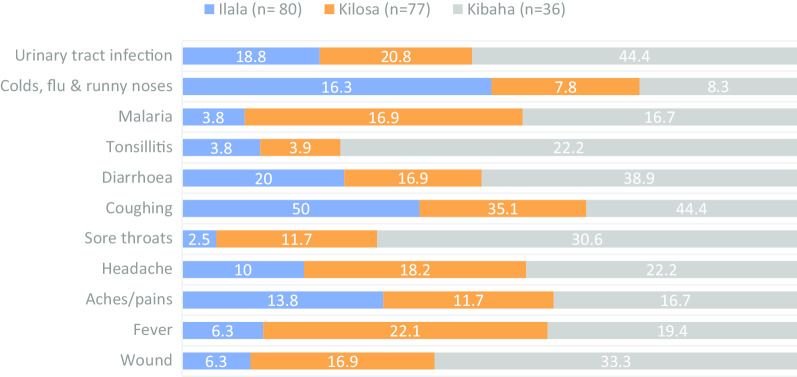
Distribution of the proportion of disease conditions treated in humans by self-medication by district

Out of 205 participants who reported to have practised self-medication, 193 (94.1%) could recall the health conditions they were suffering from. The overall frequency of self-medication by a disease condition in the decreasing order were coughing (43.0%), urinary tract infection (24.4%), diarrhoea (22.3%), wound (15.5%), headache (15.5%), fever (15.0%), aches/pains (13.5%), sore throats (11.4%), malaria (11.4%), colds, flu and runny noses (11.4%) and tonsillitis (7.3%). The largest proportion of individuals who had practised self-medication based on these conditions was consistently observed in Kibaha. However, coughing that was most frequently reported condition in Ilala, and colds, flu and runny noses, malaria and fever that were mainly reported by participants from Kilosa (Fig. 8).

Following self-medication, almost two-thirds (64.3%) of participants reported having recovered from illness. However, about one-third (31.6%) reported some improvement while 3.6% reported no change and 0.5% had their situation worsened. In the absence of favourable outcome following self-medication, 9.8% of participants (*n* = 193) took no action while 90.2% took some actions, which included (*n* = 174) seeking care from a healthcare facility (16.1%), repeating self-medication (12, 6%), visiting traditional healer (3.5 %) and/or seeking care from spiritual healers (0.6%). There was insufficient statistical evidence for sociodemographic factors to influence these practices (*p* > 0.05).

### Association between sociodemographic variables and knowledge, attitude and practices

The results of bivariate linear regressions analysis showed that compared with participants from Kibaha, the scores of knowledge of antimicrobials were significantly lower by 0.49 and 0.45 among participants from Kilosa and Ilala, respectively. The antimicrobial knowledge scores tended to increase with an increased level of education. Participants with college/university (β =1.93; 95% CI: 1.21, 2.65), secondary (β= 0.99; 95% CI: 0.54, 1.44) and primary (β =0.72; 95% CI: 0.35, 1.09) education had significantly higher level than those with no formal education. For each unit increase of age, there was an increase of 0.03 (95% CI: 0.01, 0.05) of AMR knowledge scores. High AMR knowledge scores were significantly associated with being a male (β=1.16; 95% CI: 0.41, 1.90), having college/university education (β=3.69; 95% CI: 1.94, 5.45), secondary education (β=3.69; 95% CI: 1.94, 5.45) and petty trading as the main source of income (β=1.17; 95% CI: 0.45, 1.88). Compared with those from Kibaha, participants residing from Ilala (β=1.89; 95% CI: 1.06, 2.72) and Kilosa (β=1.51; 95% CI: 0.70, 2.33) had significantly higher scores of AMU attitude. High scores of AMU attitude were significantly associated with college/ university (β=2.09; 95% CI: 0. 50, 3.68) and primary (β=1.63; 95% CI: 0.82, 2.44) level of education.

A statistically significant association between AMR attitude scores and area of participant’s residence was recorded in Ilala (β =1.01; 95% CI: 0.32, 1.69). For each unit increase of age, there was 0.02 increase in AMR attitude scores (β =0.02; 95% CI: 0.01, 0.04). The scores were significantly associated with the participant's level of education; compared to participants who had not attained formal education, participants with college/university (β=2.43; 95% CI: 1.13, 3.74), secondary (β=1.76; 95% CI: 0.95, 2.58) and primary (β=1.42; 95% CI: 0.75, 2.09) had higher scores. Being employed was associated with low AMR attitude scores (β=-1.30; 95% CI: -2.15, -0.45) while a marginal association was observed between the scores and petty trading (β=0.54; 95% CI: 0.01, 1.08). The practices on AMU was significantly associated with an increase in participant's age (β=0.01; 95% CI: 0.001, 0.009). Being employed was significantly associated with low practice scores (β= -0.22; 95% CI: -0.43, -0.01).

Multiple linear regression analysis showed that residing in Ilala (β_adj_ =-0. 82; 95% CI: -1.24,-0.40) and Kilosa (β_adj_ =-0.42; 95% CI: -0.81, -0.03), and being married, (β_adj_ =0-0.3; 95% CI: -0.62, -0.02) were significantly associated with low scores of knowledge. On the contrary, increase in the level of education (university/college, β_adj_ =2.48; 95% CI: 1.70, 3.25, secondary, β_adj_ = 1.49; 95% CI: 0.95, 2.03, primary, β_adj_=1.07; 95% CI: 0.66, 1.49) was significantly associated with increased AMU knowledge scores. The factors that were associated with high scores of AMR knowledge were old age (β_adj_ =0.04, 95% CI: 0.02, 0.07), being a male (β_adj_=0.09; 95% CI: 0.12, 1.65) and having attained college/university (β_adj_=3.28; 95% CI: 1.39, 5.17) and secondary (β_adj_=1.56; 95% CI: 0.25, 2.87) education. Compared with participants from Kibaha, significantly higher scores of AMU attitudes were recorded among participants from Ilala (β_adj_=1.91; 95% CI: 0.98, 2.85) and Kilosa (β_adj_=1.56; 95% CI: 0.70, 2.43). College/university (β_adj_=1.89; 95% CI: 0.18, 3.60) and primary (β_adj_=1.87; 95% CI: 0.95, 2.79) levels of education were significantly associated with AMU attitude score gain. Each unit increase in the participant’s age had a significant influence on AMR attitude scores (β_adj_=0.04; 95% CI: 0.02, 0.06). Having attained college/university (β_adj_=2.21; 95% CI: 0.82, 3.61), secondary (β_adj_=2.00; 95% CI: 1.02, 2.97), or primary (β_adj_=1.49; 95% CI: 0.74, 2.24) education was significantly associated with increased AMR attitude scores. The AMU practice score gains was influenced by participant’s level of education; compared to those who had not attained formal education, those with college/university (β_adj_=0.35; 95% CI: 0.07, 0.63), secondary (β_adj_=0. 24; 95% CI: 0.05, 0.44), or primary (β_adj_=0.19; 95% CI: 0.04, 0.34) education had higher scores. The overall increase in KAP scores were significantly influenced by increased participant’s age (β_adj_= 0.10; 95% CI: 0.05, 0.15) and increased level of education; primary (β_adj_=5.32; 95% CI: 3.27, 7.37), secondary (β_adj_=6.18; 95% CI: 3.53, 8.84) and college/university (β_adj_=9.85; 95% CI: 6.04, 13.67).

**Fig. 9:**
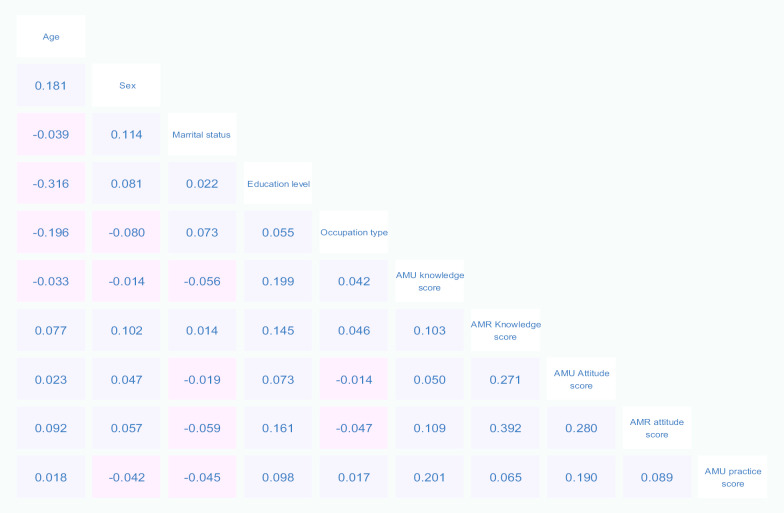
Heatmap matrix plot of pairwise correlations between socio-demographic variables and each of KAP domain scores (Positive correlations (rho > 0) show in blue and negative correlations (rho < 0) in pink)

The greatest positive correlations of the variables were observed between AMU knowledge scores and AMU practice scores (rho =0.201); AMR knowledge scores and AMU attitude scores (rho =0.271); AMU attitude scores and AMR attitude scores (rho =0.280); and AMR knowledge scores and AMR attitude score (rho =0.392). The greatest negative correlation was observed between age and education level (rho =-0.316) (Fig. 9).

### Association between KAP scores

The results of linear regression analysis of the association between KAP scores showed that each unit increase in AMU knowledge score was associated with 0.06 increase in the AMU practice scores (β= 0.06; 95% CI: 0.04, 0.09). For each unit increase of AMR knowledge score, there was 0.21 increase in the scores of AMR attitude (β=0.21; 95% CI: 0.16, 0.25). Each unit increase on AMU attitude scores was associated with a 0.01 increase in the AMU practice scores (β=0.01; 95% CI: 0.01, 0.02). The results showed further that AMU knowledge scores (β_adj_=0.06; 95% CI: 0.03, 0.08) and AMU attitude scores (β_adj_=0.01; 95% CI: 0.01, 0.03) together had a better influence on the increase in AMU practice scores than their independent effect.

## Discussion

Inappropriate AMU and associated risk of AMR is an increasing public health problem globally [37] with potentially devastating consequences in LMIC_S_ [22, 23]. The results of this study suggest that there is a high level of awareness regarding antimicrobials and their use for disease management in humans in the three districts selected for this study. However, there is insufficient knowledge about AMU and AMR. The level of awareness observed in our study regarding antimicrobials and their use is similar to the findings reported by studies in Nepal and Jordan [38, 39]. The observed poor knowledge regarding AMU and AMR accords with the findings reported previously [39, 40]. The pattern observed in our study for knowledge of AMU and AMR to increase with an increased level of participants' education, contrasts with the findings of previous studies elsewhere [39–42]. Overall, over half of the participants were not aware of AMR and the knowledge level was considered moderate, similar to the finding reported in Nepal [39]. However, the level observed in our study is lower than that reported in Norway [43]. From the literature, quite a few studies have explored the knowledge of the general population on AMR. Most have focused on specific groups of individuals such as professionals and students [42, 44–46]. A recent systematic review [47] has indicated that only a small proportion of participants in Europe (43%), Asia (26%) and North America (22%) have ever heard of AMR.

We found that AMU was a common practice and amoxicillin was the most preferred antimicrobial. The findings correspond to those reported in Ethiopia [41], Uganda [48], and Asia [39]. Based on the fact that that increased frequency of AMU is a significant driver of AMR, especially when they are used inappropriately [49–51], it is plausible to suggest that these most used antimicrobials are at increased risk of resistance. For instance, amoxicillin use has been strongly associated with increased carriage of amoxicillin-resistant bacteria [52] and high multidrug resistance has been reported in a recent systematic review [51]. Given the low rate of development of new generations of antimicrobials, rational use of existing ones is critical for the long-term availability of effective treatment of infections [42, 43]. The pathogen-specific epidemiological models of AMR have been proposed for community-acquired infections [53] and the volume of drug use has been identified as the major selection pressure driving changes in the frequency of AMR in the community [54].

The frequent sources of antimicrobials reported were the healthcare facility and pharmacies/basic drugs shop, which is similar to the findings reported elsewhere [10, 39, 55]. Retail pharmacies have emerged as the primary level of outpatient care in Africa leading to use of antimicrobials without consultation, confirmatory diagnosis or prescription [16, 17]. Pharmacies [55] and healthcare facilities [56, 57] have been reported as the most common sources of antimicrobials associated with increased use in the community. In most low- and middle-income countries (LMIC), antimicrobials can be obtained from such sources without medical prescriptions [14]. Previous studies in Tanzania reported a high rate of non-prescription dispensing of antimicrobials from the pharmacies [55, 58]. It has been suggested elsewhere that physicians may attempt to meet a patient's expectation even though they feel antimicrobials are unnecessary [59]. The health care facilities in most of LMIC are characterized with inadequate laboratory diagnostic capacity and the high clinician-patient ratio [60, 61]. There is often no evidence-based decision in the course of treatment, and the clinicians are overwhelmed and there is frequently an inadequate time for sufficient education and communication with the patient on adherence to the treatment regimen and its importance [23]. It seems that the practice of obtaining antimicrobials from pharmacies/basic drugs shops without prescription or by the participant’s pressure to influence prescription together with low knowledge may elevate inappropriate use of antimicrobials leading to the potential risk for AMR. Pharmacies appear to be more accessible to the public as they are characterized by shorter waiting time, free consultation and negotiations on treatment options based on the financial capability of the patients [16].

In our study, we recorded a moderate attitude regarding the appropriate use of antimicrobial, which was a lower level than previously reported by other studies in Tanzania [40] and in Hong Kong [62]. However, the different scoring schemes used between our study and these other studies might have led to the different conclusions made regarding the attitude scores. For instance, whereas in our study the observed score of 72.7% was considered moderate, the study by Mbwambo and others [40] reported a score of 62.7% that was regarded as good knowledge of antimicrobials use. A study in Hong Kong [62] found the level of 77.0% that was considered an adequate knowledge of antimicrobial use. Individual’s attitude has been reported to influence antimicrobial use practices [63–66]. The observed level of knowledge and attitude may have influenced the AMU practice documented in the study districts as it has been suggested elsewhere [40, 67, 68]. Being of old age and with an increased level of education were found to influence KAP scores, the observation which contrasts with other studies [40, 42, 69]. In our study, the practices of self-medication were more frequent among men than women. Studies elsewhere have also reported that self-medication with antimicrobials is associated with male gender [70]. The prevalence of self-medication in this study was 23.6%, which is lower than that reported previously in Tanzania [55, 71]. In a recent systematic review, a prevalence of 7.3% to 85.6% self-medication of antimicrobial was reported in South-East Asia countries [72]. Self-medication with antimicrobial has been described as one of the major factors contributing to drug resistance [51, 73]. The problem of self-medication is a common practice in LMIC including Tanzania. Results from previous studies in Kilosa and Moshi districts in Tanzania showed that the guardians/parents practice self-medication and once their children get sick, they preferred to purchase drugs from the pharmacies /drug shops or use left-over medicines from their homes, neighbours, relatives or friends and other sought advice from vendors [74, 75].

The observed inadequate knowledge of AMU and AMR, inappropriate attitude and practices should be considered as alarming problems and potential signals for the occurrence of AMR that require immediate action. Policy and decision-makers could make use of the evidence generated by this study as one of the inputs to reinforce the medicine use policy and guidelines to mitigate the risk of AMR. Our findings call for tailored interventions such as education programmes targeting the community regarding appropriate use antimicrobial and appropriate ways to prevent AMR. Specific groups should be targeted for education programmes, such as those with a lower level of formal education, who have demonstrated less knowledge and inappropriate attitudes and poor practices to antimicrobial use and measures against their resistance.

## Limitations

When interpreting the findings from this study, it is important to note some of its limitations. First, the selection of a limited number of study regions, districts and wards was based largely on a purposive selection process. This means that it will be difficult to draw generalizable inferences from the results obtained in the few areas. However, the findings could apply to areas with similar settings. Second, the quality of some of the interview-based data might have been affected by recall bias and decision to share desirable and undesirable practices amongst the participants. Third, the role of other potentially relevant explanatory factors was not ruled out in our analysis. For instance, household structure (including family size and age distribution) and economic (wealth) status were not considered in this study. Socio-demographic variables were limited to only the respondent who represented the household. Inclusion of other potential parameters might have improved further our understanding regarding AMU and AMR in the study areas. Fourth, because of the cross-sectional nature of the study, it was not possible to establish causal inferences between knowledge, attitudes and practices. Besides its limitations, this study provides important information for targeted mitigation measures to address challenges related to the inappropriate use of antimicrobials and AMR in the study areas. The generated data could save as baseline or reference for future research and monitor the efficiency of future interventions.

## Conclusion

This study has provided information on the levels of knowledge of AMU and AMR, sources, attitudes and AMU practices in Ilala, Kibaha and Kilosa districts. The study documented a moderate level of knowledge, attitudes and practices regarding AMU and AMR in the districts. The results suggest that old age and high level of education together had a positive influence on the knowledge, attitudes and practices regarding AMU and AMR. The correlation between knowledge and attitudes was moderate; and poor between knowledge and practices; and attitudes and practices. The findings are important to guide policy formulation, planning and implementation of AMU and AMR related programmes, especially those targeting community-based awareness, education and sensitization.

## Data Availability

The datasets generated and/or analysed during the current study are available in the Muhumbili University of Health and Allied Sciences (MUHAS) in Dar es Salaam, Tanzania and is available on reasonable request.

## Abbreviations

AMU: Antimicrobial use
AMR: Antimicrobial resistance
KAP: Knowledge, attitudes and practices
LMICS: Low and middle-income countries
IQR: Interquartile range
β: Regression coefficient beta
βadj: Adjusted coefficient beta
95% CI: 95 percent confidence intervals
%: Percentage
rho: Spearman rank-order correlation coefficient
